# Seroprevalence of the Serological Markers of Transfusion-Transmissible Infections among Volunteer Blood Donors of Kosti Obstetrics and Gynecology Hospital

**DOI:** 10.3390/medicines8110064

**Published:** 2021-10-29

**Authors:** Babiker Saad Almugadam, Omer Mohammed Ali Ibrahim, Yousif Mousa Alobaid Ahmed

**Affiliations:** Department of Microbiology, Faculty of Medical Laboratory Sciences, University of El Imam El Mahdi, Kosti 11588, Sudan; omermi2014@gmail.com (O.M.A.I.); abuwaddah2016@gmail.com (Y.M.A.A.)

**Keywords:** anti-HIV1/2, anti-HCV, anti-*T.pallidum*, HBsAg, TTIs

## Abstract

Background: Transfusion-transmissible infections are well-known global health challenges. The present study is proposed to investigate the seropositivity of anti-HIV1/2, anti-HCV, HBsAg, and anti-*T.pallidum* among volunteer blood donors of Kosti Obstetrics and Gynecology Hospital. Methods: Our study was conducted in a cross-sectional retrospective manner. The data of donors who attended Kosti Obstetrics and Gynecology Hospital throughout 2016 to 2018 were reviewed and retrieved manually from blood bank records. Results: Out of 8139 donors, 22.52% were seropositive for serological markers of TTIs and 1.67% were seropositive for at least two serological markers of TTIs. The overall seropositivity rate of anti-HIV1/2, HBsAg, anti-HCV, and anti-*T.pallidum* was 1.77%, 6.07%, 1.14%, and 11.87%, respectively (*p* < 0.000). Anti-*T.pallidum* was the most frequently detected (*p* < 0.05) marker across all study variables. TTIs seroprevalence was significantly (*p* < 0.05) varied according to the age, residence, occupations, and blood groups. Notably, there was a rising trend in the rate of anti-HIV1/2 and seropositivity for more than one marker with age (*p* < 0.000). Regionally, rural area residents had a higher rate of anti-HIV1/2 (2.20%), HBsAg (6.31%), anti-HCV (1.42%), anti-*T.pallidum* (18.38%), and multiple markers seropositivity (2.28%) compared to urban areas. Between occupations, the highest rate of anti-HIV1/2 (*p* = 0.483), HBsAg (*p* = 0.003), anti-HCV (*p* = 0.408), anti-*T.pallidum* (*p* < 0.000), and multiple markers seropositivity (*p* < 0.000) were detected in farmers. Regarding the screening, we also found that the frequency of anti-*T.pallidum* was significantly (*p* = 0.003) higher in donors who carry the AB+ve blood group, whereas anti-HCV (1.83%) was more frequent in donors carry O−ve blood group (*p* = 0.255). As seen, anti-*T.pallidum*+HBsAg was the most frequently (1.22%) co-occurring markers. In contrast, anti-*T.pallidum*+anti-HIV1/2+HBsAg was the lowest frequency one (*p* < 0.000). Conclusions: The study showed an alarming rate of TTIs, which suggests the requirement for comprehensive surveillance and health education programs.

## 1. Introduction

Human immunodeficiency virus (HIV), hepatitis C virus (HCV), hepatitis B virus (HBV), and *T.pallidum* are potentially dangerous infectious pathogens that may be acquired through blood transfusion [1,2]. At a global level, T.pallidum affects approximately six million people aged 15–49 years annually [2]. In 2017, 36.8 million of the world population lives with HIV, and 1.94 million are new cases [3]. As reported in the study of Petruzziello et al. [4], the global prevalence of HCV is 2.5%. Furthermore, it has been estimated that around one-third of the world’s population was infected by HCV or HBV in 2015. Approximately 6.1% of the African populations have HBV infection [5]. According to the World Health Organization (WHO), five million cases of acute hepatitis B occur every year [6]. On the other hand, approximately five percent of HBV-infected populations are in a chronic carrier state [5].

Annually, the transfusion of blood and its products save a lot of lives [7]. More than 81 million blood units are collected globally every year, of which, 18 million units are not tested for transfusion-transmissible infections (TTIs) [8]. Thus, a huge number of the world population are at risk of exposure to chronic and life-threatening illnesses, such as HBV, HCV, or HIV infections, attributable to the transfusion of unsafe blood or its products [7]. Long-lasting HIV, HBV, or HCV infections increase the risk of morbidity and mortality as a result of the effects of the chronic carrier state [3,5,6]. For example, chronic HBV or HCV infections may lead to liver cirrhosis and failure, hepatocellular carcinoma, and death [5]. To be exact, around 15–40% of chronically HBV-infected patients will progress into liver cirrhosis, failure, or cancer, and 15–25% will eventually die. Every year, approximately 800,000 individuals die due to hepatitis B-related liver cirrhosis and cancer [6]. In 2010, HBV was ranked as the 15th cause of death globally [6]. Moreover, HCV and HBV are accountable for around ninety percent of viral hepatitis mortality, which was estimated to be around 1.4 million deaths every year [5]. Globally, 0.95 million deaths were caused by HIV in 2017 [3]. Notably, the global practice of selecting a clinically fit blood donor, along with effective screening tests, has significantly declined the risk of post-TTIs [9].

In Sudan, the magnitude of HIV, HBV, HCV, and *T.pallidum* infections are still indistinct. Many studies had been performed to recognize the epidemiology of these illnesses. Based on a systemic review study, 12.07% is a positivity rate of HBV, whereas the pooled prevalence of HIV and HCV antibodies was 1% and 2.74%, respectively [1]. The aforementioned emphasizes the need for regular surveillance and control programs to limit the spread and complications of such infections. Due to the lack of highly accurate diagnostic tests (such as nucleic acid methods) for TTIs in many regions and countries, such as Sudan, rapid diagnostic tests (non-confirmatory) were commonly used for the screening of TTIs [10,11]. The assessment and screening of the frequency of HCV, HBV, HIV, and *T.pallidum* infections among blood donors are valuable in understanding the epidemiology of TTIs, and it is also a matter of concern to ensure safe blood transfusion. The current study intended to screen the seropositivity of anti-HIV1/2, anti-HCV, HBsAg, and anti-*T.pallidum* among blood donors of Kosti Obstetrics and Gynecology Hospital.

## 2. Methods

### 2.1. Study Design, Duration, and Area

This was a cross-sectional retrospective study carried out at the blood bank of Kosti Obstetrics and Gynecology Hospital to assemble data of blood donors from 1 January 2016 to 31 December 2018. Kosti Obstetrics and Gynecology Hospital is located in Kosti city. It is a regional tertiary-care hospital.

### 2.2. Ethical Consideration

An ethics clearance needed in order to conduct the study was granted by the Ethics Review Committee of Kosti Obstetrics and Gynecology Hospital. The rules of the declaration of Helsinki concerning human research were applied. Volunteer participant’s privacy was guaranteed by excluding and coding the identity of donors, and no names were involved in the data collection and analysis. Donor’s informed consents were not obtained since the study did not directly involve donors, but was instead based on data obtained from blood bank records, and it is impossible to track back the blood donors for agreement.

### 2.3. Study Subjects and Data

The study population includes all volunteer blood donors who attended Kosti Obstetrics and Gynecology Hospital and were recruited from 1 January 2016 to 31 December 2018. All blood donors in the study area are non-remunerated, and most were relatives of the blood receiver. The blood donor’s selection was based on a pre-donation examination that included health history, along with physical and laboratory checkups. Donor’s eligibility criteria were age ranged 18–65 years, hemoglobin (Hb) ≥ 13.5 g/dL in males or ≥12.5 g/dL in females, bodyweight ≥ 45 Kg, normal blood pressure, and absence of clinical illnesses. Disqualified individuals for blood donation include those with abnormal blood pressure, Hb < 13.5 g/dL (for males) or <12.5 g/dL (for females), jaundice, infection, age < 18 or over 65 years, bodyweight < 45 Kg, hypertension or current fever, or recent history of donation of less than six months. Malnourished people, pregnant or breastfeeding women, as well as individuals with a recent history of chronic or venereal disease, surgery, asthma, or clinical sign of illness, were excluded. Before donation, each individual has completed the required (mandatory) blood donation information. In this study, socio-demographic information (gender, residence, age, and occupation), along with transfusion-transmissible infections screening (anti-HIV1/2, HBsAg, anti-HCV, and anti-*T.pallidum*) and blood grouping tests results, were reviewed and retrieved manually from blood bank records for each blood donor. A checklist with the required variables was adopted to extract data from blood bank records.

### 2.4. Screening of Anti-HIV1/2, HBsAg, Anti-HCV, and Anti-T.pallidum

A venous blood sample was collected from each eligible donor and divided into plain (without anticoagulant) and EDTA blood containers. All of the collected samples were processed immediately. Blood samples of EDTA containers were used to investigate the blood group and Rhesus factor by slide method of blood grouping using monoclonal antisera (Fortress Diagnostics, United Kingdom). For screening of TTIs, the plain container (without anticoagulant) blood samples were allowed to clot at room temperature, and then serum was separated by centrifugation at 3000 rpm for 5 min. Next, serum samples were subjected to anti-HIV1/2, anti-HCV, HBsAg, and anti-*T.pallidum* screening by rapid chromatographic immunoassay test kits (ACON^®^ Laboratories, Inc. Sang Diego, CA, USA). All of the tests protocols were carried out according to the manufacturer’s instructions. Briefly, the tests kits were brought to room temperature, and then the determined volume of serum sample was added into the sample well of each cassette. Additionally, one drop of the HIV and HCV buffer was added into the buffer well of HIV and HCV rapid test cassettes, respectively. The results reading and interpretation were carried out according to kits’ guidelines. Each HIV or syphilis rapid test cassette kit has a >99.9% and 99.3% relative sensitivity and specificity, respectively. The relative diagnostic sensitivity and specificity of the HBsAg rapid test cassette were 99.4% and 99.5%, respectively. HCV rapid test cassette has 98.1% sensitivity and 98.9% specificity.

### 2.5. Statistical Analysis

Study data were collected onto data sheets and then inputted and analyzed using statistical package for social sciences (SPSS) software version 21. The frequencies of TTIs were presented in numbers and percentages. Pearson’s chi-squared and Fisher’s exact tests were used to test the variation between categorical data at a 95% level of significance. A *p* value of less than 0.05 was reported as statistically significant.

## 3. Results

### 3.1. Socio-Demographic Characteristics of Blood Donors

Data of 8139 blood donors recruited in the blood bank of Kosti Obstetrics and Gynecology Hospital throughout 2016 to 2018 were checked and retrieved manually from records. Demographic characteristics of blood donors are presented in Table 1. A total of 25.42% (2069), 38.48% (3132), and 36.10% (2938) of the total blood donors were recruited during 2016, 2017, and 2018, respectively. More than half of the donors (54.53%) were in the age range of 18–27 years. People living in urban areas constitute 52.68% of participants. A free business is the major stated job by 76.60% of study subjects. Moreover, the vast majority of donors (67.70%) were reported as O+ve (Table 1).

### 3.2. Seroprevalence of Anti-HIV1/2, Anti-HCV, HBsAg, and Anti-T.pallidum

A total of 22.52% of donors were seropositive for TTIs. The annual seropositivity rate of TTIs was comparatively highest in 2018 (24.98%) and lowest in 2016 (19.91%), *p* < 0.000. The overall rate of anti-HIV1/2, HBsAg, anti-HCV, and anti-*T.pallidum* was significantly (*p* < 0.000) different among donors. Indeed, anti-*T.pallidum* was the most frequently detected one, followed by HBsAg (6.07%). On the other hand, the annual percentage of those who tested positive for anti-HIV1/2, anti-HCV, HBsAg, and anti-*T.pallidum* was varied across the study periods (2016, 2017, 2018). Notably, anti-*T.pallidum* was the most prevalent one across all periods, followed by HBsAg. The rate of anti-HIV1/2 (*p* < 0.000) and anti-*T.pallidum* (*p* = 0.027), and the co-occurrence of the serologic markers of co-infections (*p* < 0.000) were also significantly different among study periods (Table 2).

**Figure 1:**
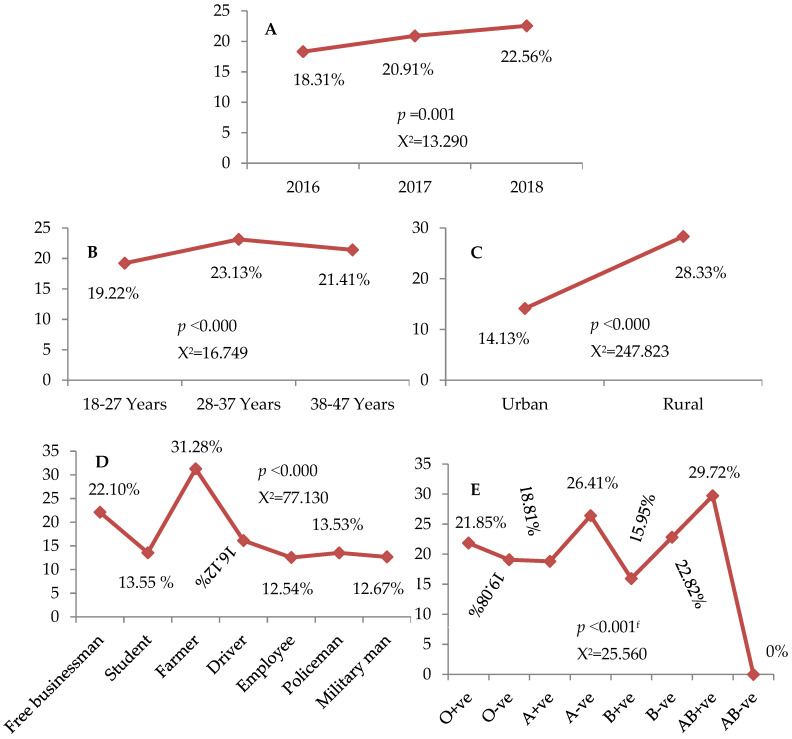
(**A**–**E**): Trend of the single serologic marker. (**A**): Throughout 2016 to 2018, (**B**): among age groups, (**C**): according to the residence, (**D**): in relation to occupations, (**E**): among blood groups and Rhesus factor. X^2^: chi-squared. Data assessed by the Pearson chi-squared and Fisher’s exact tests^f^.

Out of 8139 donors, 20.85% (1697) were seropositive for only a single type of the investigated TTIs (Supplementary Figure S1). Figure 1A–E displays the trend of the single serologic marker seropositivity among blood donors. Notably, the percentage of donors who showed a positive result for a single marker (one type of TTIs) was significantly different across study periods (X^2^ = 13.290, *p* = 0.001), as well as the age groups (X^2^ = 16.749, *p* < 0.000), residence (X^2^ = 247.823, *p* < 0.000), occupations (X^2^ = 77.130, *p* < 0.000), and blood groups (X^2^ = 25.560, *p* < 0.001) of blood donors (Figure 1A–E). Indeed, a significantly higher rate (22.56%) of positive results reported for a single marker was seen in donors recruited during 2018 (Figure 1A). Individuals in the age range of 28–37 years (23.13%) and rural areas (28.33%), as well as of the AB+ve blood group (29.72%) showed the highest rate for single marker seropositivity (Figure 1B,C,E). Furthermore, we found that farmers had the highest rate of seropositivity (X^2^ = 77.130, *p* < 0.000) for a single serologic marker 31.28% (Figure 1D).

As shown in Table 3, the TTIs markers seroprevalence significantly (*p* < 0.000) differed according to the age, residence, occupations, and blood groups (Table 3). A rising trend in the seropositivity rate of the anti-HIV1/2 and a serologic sign of multiple infections with age has been observed (*p* < 0.000). Anti-*T.pallidum* was the most frequently detected (*p* < 0.05) marker among all study variables. Among age groups, anti-HCV was the significantly lowest frequently detected marker. Excluding anti-*T.pallidum*, the proportions of the markers seropositivity were statistically varied (*p* < 0.000) among age groups (Table 3).

Regionally, rural areas residents had a higher rate of anti-HIV1/2 (2.20%), HBsAg (6.31%), anti-HCV (1.42%), and anti-*T.pallidum* (18.38%) seropositivity compared to urban areas. The co-occurrence of multiple markers (a serologic sign of multiple infections) was also more present in donors of rural (2.28%) than urban (1.11%) areas, *p* < 0.05. Moreover, anti-*T.pallidum* was the most frequently detected marker in both rural and urban areas. In contrast, anti-HCV was the lowest detected marker (Table 3).

Between occupations, the seropositivity of the serologic sign (anti-HIV1/2, HBsAg, anti-HCV, anti-*T.pallidum*, and multiple markers) of all TTIs was significantly dissimilar (*p* < 0.000). Farmers had the highest rate of seropositivity for anti-HIV1/2 (*p* = 0.483), HBsAg (*p* = 0.003), anti-HCV (*p* = 0.408), anti-*T.pallidum* (*p* < 0.000), and a serologic sign of multiple infections (*p* < 0.000). In contrast, employee donors had the lowest rate of anti-HIV1/2 and anti-HCV. Likewise, the military men showed the lowest rate of anti-*T.pallidum* (5.63%) and multiple markers (0.70%) seropositivity (*p* < 0.000). The lowest positivity rate for HBsAg (2.64%) was detected in (*p* < 0.003) students (Table 3).

The link of blood groups and Rhesus factor with the seroprevalence of the serologic markers of TTIs was also analyzed. Regarding the screening, we found that the frequency of anti-HIV1/2 was higher (4.40%) in donors of the A−ve blood group (*p* = 0.238). The anti-*T.pallidum* (*p* = 0.004) and co-occurrence of more than a single marker (*p* = 0.519) were more present in donors of the AB+ve blood group. Moreover, individuals who carry the B−ve blood group had the highest rate (9.78%) of HBsAg (*p* = 0.256). A slight peak rate (*p* = 0.255) of anti-HCV (1.83%) was detected in donors of the O−ve blood group (Table 3).

### 3.3. Co-Occurrence of Multiple Markers (Serologic Sign of Multiple Infections)

Out of 8139 donors, 1.67% (136) had a serologic sign of multiple infections (Table 2). Among blood donors (8139), anti-*T.pallidum*+HBsAg was the most frequently (1.22%) co-occurring serologic markers of multiple infections, followed by anti-*T.pallidum*+anti-HIV1/2 (0.13%), whereas, ant-*T.pallidum*+anti-HIV1/2+HBsAg was the lowest frequency one, *p* < 0.000. Of 136 individuals with seropositivity for more than one serologic marker, 73.53% (100) were seropositive (*p* < 0.000) for anti-*T.pallidum*+HBsAg (Table 4).

## 4. Discussion

Transfusion-transmissible infections are well-known global health challenges. Blood screening ensures the safety of transfusion and declines the risk of TTIs. In the present study, all blood donors were males. This is consistent with previous local studies [11,12,13,14]. Globally, the published data [8,15,16,17,18,19,20,21,22,23,24,25,26,27,28,29,30,31,32,33,34] also indicated the low contribution of females in blood donations compared to males, which may result from numerous aspects, including pregnancy and menstruation. In Sudan, this could also partly be related to the culture of the community. Unlike our study, Cao et al. [35] and Song et al. [36] studies reported that the majority of blood donors were females. Interestingly, all of the recruited blood donors of the present study were volunteering non-paid, which could reflect the awareness of the population regarding blood donation. In line with previous studies [8,15,17,18,19,21,25,27,28,29], we found that the majority of donors were in the age range of 18–27 years. Unlike our study, some studies reported different findings regarding the age and donors relationship [11,16,30,34]. In our work, most of the participants were recruited from urban areas. This is analogous to the previous reports from Pakistan [15], Eritrea [16], and Ethiopia [25,28]. In agreement with Arshad et al. [15], a free business is a job of more than half (76.60%) of the present study subjects. Dissimilar to our result, Bdella et al. [21] and Abebe et al. [25] stated students as major blood donors, and Heyredin et al. [28] presented employees as main blood donors and farmers as the lowest frequency one. Regarding blood type analysis, O+ve was detected in 67.70% (majority) of the current study donors. In this regard, a similar finding has been reported previously [22,34].

Regarding the screening, the overall seropositive rate of TTIs was 22.52%, which is higher than previous studies [8,15,16,17,19,20,21,22,27,28,29,30,31,32,33,35,36,37,38]. It is also higher than studies performed in other states of Sudan [13,34,39], as well as a study [11] conducted in our study area formerly. In contrast, it is lower than the study result of Hang et al. [18]. In this study, the seroprevalence of anti-HIV1/2, HBsAg, anti-HCV, and anti-*T.pallidum* was 1.77%, 6.07%, 1.14%, and 11.87%, respectively. These findings were higher than those obtained in many previous studies [13,39] in Sudan. Comparatively, several studies from all over the world also reported a low seropositivity rate for anti-HIV1/2 [15,16,17,19,20,21,22,26,29,31,32,33,35,36,37,38], anti-*T.pallidum* [8,15,16,17,19,20,21,22,26,27,29,30,31,32,33,35,36,37,38], and both HBsAg and anti-HCV [16,17,19,20,21,24,29,30,32,33,36,37,38] compared to the current study. In our area (Kosti locality), many studies concerning TTIs have been performed [11,12]. In this regard, a study carried out throughout January to April 2014 showed a lower rate of anti-HIV1/2 and anti-*T.pallidum* compared to the present study [12]. Likewise, in another study [11], the rate of HBsAg, anti-HCV, and anti-*T.pallidum* was lowered, but anti-HIV1/2 was higher than the present result. On the other hand, studies carried out in Angola from 2011 to 2016 [23] and North Darfur State (Sudan) from 2017 to 2019 [14] demonstrated a higher rate of anti-*T.pallidum* compared to our findings. Furthermore, another study conducted in Port Sudan (Sudan) showed a higher level of HBsAg but a lower rate of anti-HCV, anti-HIV1/2, and anti-*T.pallidum* compared to our result [34]. According to a systemic review study [1], our reported rate of HBsAg and anti-HIV1/2 was lower, but the rate of anti-HCV was more than the estimated pooled prevalence’s rate in Sudan. In this study, the percentage of anti-HIV1/2 was lower when compared with the studies of Jary et al. [27] and Adu-Poku et al. [8]. In Chinese blood donors in the mainland, the pooled prevalence of HCV infection was higher than in our study. Relatively, Cao et al. [35] and Jary et al. [27] reported a higher rate of HBsAg and anti-HCV compared to the present result. However,, Arshad et al. [15], Abebe et al. [25], Adu-Poku et al. [8], and Hroob et al. [22] showed a lower HBsAg and higher anti-HCV rate compared to this study. In contrast, Hasan et al. [31] showed a higher level of HBsAg but a lower rate of anti-HCV in male blood donors compared to our result. Warningly, we observed a substantial rise (*p <* 0.05) in the annual rate of TTIs and anti-*T.pallidum*, which is an extremely alarming condition. Similarly, a study conducted in North Darfur State (Sudan) reported a significant increase in the seroprevalence of syphilis all over the study duration [14]. Notably, the HBsAg seropositivity had also risen throughout the current study duration, but it is not significant. According to a previous study, the annual seropositive rate of TTIs, HBsAg, and anti-*T.pallidum* was significantly varied across study times [16]. A study carried out at Dongola Specialized Hospital (Sudan) also presented a mild difference in the annual rate of HBV, HCV, HIV, and syphilis [13]. Comparable with the study of Keleta et al. [16], anti-*T.pallidum* was the most frequent marker across all our study periods (*p <* 0.05). Although the exact reasons underlying the discrepancies between studies are indistinct, perhaps this might be due to the variation in geographical location, epidemic nature of diseases, study population and sample size, and screening tests. Collectively, these findings indicated the need for comprehensive surveillance and health education programs to decrease the burden and risk of transfusion-transmissible infections.

In this study, the overall rate of TTIs was significantly (*p <* 0.000) varied among age, residence, jobs, and blood types. Within the study variables, anti-*T.pallidum* was the most frequently detected marker. Interestingly, the overall rate of TTIs, anti-HIV1/2, and a serologic sign of multiple infections was significantly raised with age. In age groups, the most and least frequently detected marker was anti-*T.pallidum* and anti-HCV, respectively. Analogous to our result, Keleta et al. found that the overall rate of TTIs and anti-*T.pallidum* significantly increased with age [16]. In line with current study findings, Arshad et al. [15] reported a high rate of TTIs in those more than 35 years old compared to the age ranges of 18–25 or 26–35. Formerly, a study carried out in Ethiopia reported that the rates of HBV and HCV infections were higher in ages of 46–65 [25]. In males, Jary et al. found that HIV seroprevalence significantly increased with age [27]. Comparable to our findings, Heyredin et al. [28] found that there were more TTIs in the 46–65 age group, followed by the 31–45 age group, and then the 18–30 age group. Likewise, Quintas et al. also observed that the rate of syphilis was significantly higher in individuals aged ≥ 40 years old, followed by those aged 35–39 years old [23]. Analogous to the study of Ahmed et al. [11] that was conducted in our area, we observed that the HBsAg and anti-HCV seropositivity was significantly greater in the 28–37 age group compared to others, whereas anti-HIV1/2 seropositivity is greater in the 38–47 age group (*p* < 0.000), whereas anti-*T.pallidum* is greater in the 18–27 age group but it is not significant (*p >* 0.05). Previously, Adu-Poku et al. [8] and Hang et al. [18] reported dissimilar findings regarding age. Unlike our study, Bazie et al. [12] found that the seroprevalences of anti-HIV1/2 and anti-*T.pallidum* were higher in 20–40 years old donors than 41–55, and Mohammed et al. [34] reported that the rate of TTIs was higher in individuals aged 26–35 years, followed by those more than 35 years old. Regionally, we found that donors living in rural areas had a significantly higher rate of TTIs, anti-HIV1/2, anti-HCV, anti-*T.pallidum*, and multiple markers (a sign of multiple infections) seropositivity compared to urban areas, as well as HBsAg, but it is not significant. Previously, Keleta et al. [16] reported a higher rate of TTIs, HBV, HCV, HIV, and anti-*T.pallidum* in rural areas than urban. Likewise, a study was carried out in eastern Ethiopia also showed a higher rate of TTIs in blood donors of rural than urban areas [28]. In contrast, Arshad et al. [15] and Abebe et al. [25] showed a higher rate of TTIs and HBV, respectively, in donors who live in urban areas instead of rural areas. Analogous to our findings, Abebe et al. [25] presented a higher rate of HCV among donors of rural areas than urban areas. Concerning occupations, the current study showed that the seropositivity rate of TTIs (*p* < 0.000) or, in particular, anti-HIV1/2 (*p* = 0.483), HBsAg (*p* = 0.003), anti-HCV (*p* = 0.408), anti-*T.pallidum* (*p* < 0.000), and serologic markers of multiple infections (*p* < 0.000), was more in farmers than others. On the other hand, employee donors had the lowest rate of anti-HIV1/2, and anti-HCV (*p* > 0.05). Likewise, the military men showed the lowest rate of TTIs, anti-*T.pallidum,* and multiple markers seropositivity (*p* < 0.000), whereas the lowest positivity rate for HBsAg was detected in students (*p* = 0.003). Unlike the study of Arshad et al. [15], the rate of TTIs in the current study was lower in students than in businessmen and drivers. In line with Bdella et al. [21], we found that the overall rate of TTIs, anti-HIV1/2, and HBsAg, and the co-occurrence of multiple markers, were lower in students compared to car drivers but, in contrast, the rate of anti-HCV and anti-*T.pallidum* was higher in students than drivers. Dissimilar to our result, Hasan et al. [31] reported a high rate of HBV in students than business personnel, and Abebe et al. [25] showed a higher percentage of HBV and HCV infections in students than farmers. Comparable to Heyredin et al. [28], we observed that TTIs were highest in farmers, followed by drivers, and then students and employees. In this study, the frequency of anti-HIV1/2 was higher (4.40%) in the donors of blood group A−ve (*p* = 0.238), which is analogous to Prakash et al. [38]. Interestingly, the highest rate of TTIs (*p* < 0.000) and anti-*T.pallidum* (*p* = 0.004), in addition to the higher seropositive percentage of more than one marker (*p* = 0.519) in our study, was detected in donors of the AB+ve blood group. Similar to our findings, Hroob et al. [22] and Prakash et al. [38] found that there was a higher rate of syphilis in AB+ve blood groups than donors of other blood groups. In the present study, individuals who carry the B−ve blood group had the highest rate (9.78%) of HBsAg (*p* = 0.256), and those carry the O−ve blood group showed a peak rate (*p* = 0.255) of anti-HCV (1.83%). Previously, Hroob et al. [22] documented different findings regarding HBsAg, anti-HCV, and anti-HIV in male blood donors. Additionally, Prakash et al. [38] also showed dissimilar findings concerning the seropositive rate of TTIs, HBsAg, and anti-HCV. In line with our result, Mohammed et al. [34] reported the lowest rate of TTIs in donors who carry the AB−ve blood group, but, unlike the current result, they found that donors who carry the B−ve blood group had a higher rate of TTIs. In general, these findings highlight the TTIs and donor’s characteristic relationship.

In the current study, 1.67% (136) of 8139 donors were seropositive for more than one marker. This is relatively high when compared to previous studies that were carried out in Sudan [11,39] or other countries [15,29,35,36]. In a study [12] conducted in Kosti Teaching Hospital, there was no single case with a seropositive result for more than one serologic marker of TTIs, which is different from the current or previous [11] study outcomes. It is necessary to understand that the low numbers of donors in the study of Bazie et al. [12] may affect the interpretation of its findings. More importantly, we observed that the co-occurrence of serologic markers of multiple infections increased with age (*p* < 0.000). Likewise, it was higher in rural than urban areas, and farmers than other jobs. In line with our findings, a previous study reported [15] that the rate of multiple infections was higher in rural than urban areas, and in an age > 35 than 18–28 or 26–35 age groups. Unlike our result, a study of Ahmed et al. [11] that assessed the TTIs in the Kosti locality did not notice a significant link between the age and seropositivity of more than one marker. In agreement with current study findings, Arshad et al. [15] showed that the seroprevalence of co-infections was more in drivers than employees, and students than free businessmen, as well as being lower in students compared to employees. Moreover, we found that anti-*T.pallidum*+HBsAg was the most frequently (1.22%) co-occurred markers, and, in contrast, anti-*T.pallidum*+anti-HIV1/2+HBsAg was the lowest frequency one (*p* < 0.000). In Sudan, many previous studies [13,34,39] performed in different states reported anti-*T.pallidum*+HBsAg as the most frequent co-infection. Analogous to our result, a study performed at Xiangya Hospital (China) from 2011 to 2016 in hospitalized patients before transfusion indicated anti-*T.pallidum*+HBsAg as the most common co-infection [35]. Another study published in 2021 that covered data of 20,392 blood donors also reported anti-*T.pallidum*+HBsAg as the most frequent co-infection [32]. However, according to a previous local report [11], anti-HIV1/2+HBsAg was the most frequent co-infection in the Kosti locality (*p* > 0.05), which is inconsistent with our results. Dissimilar to our findings, a study of Jary et al. [27] reported HIV/HVB and HBV/HCV as the most common co-infections in male blood donors. Taken together, these findings showed the seropositivity of serologic markers of multiple infections among blood donors. In our study, the low sample size and use of less sensitive diagnostic methods (screening and non-confirmatory rapid tests), in addition to the lack of some data regarding the blood donors, are major limitations. Certainly, in order to minimize the risk of TTIs, it is important to strengthen the screening procedure. Moreover, the lack of some information regarding blood donors is a well-known weakness of research.

## 5. Conclusions

The current study screened the seropositivity of the serologic markers of TTIs among blood donors of Kosti Obstetrics and Gynecology Hospital and sheds light on the co-occurrence of the serologic sign of multiple infections. There was an alarming rate of anti-HIV1/2, anti-HCV, HBsAg, and anti-*T.pallidum*, which suggests the requirement for comprehensive surveillance and health education programs in order to decrease the burden and risk of transfusion-transmissible infections.

## Figures and Tables

**Table 1:** Characteristics of blood donors (*n* = 8139).

Variable		Number	Percentage
Year	2016	2069	25.42
	2017	3132	38.48
	2018	2938	36.10
Age group/Years	18–27	4438	54.53
	28–37	2982	36.64
	38–47	719	8.83
Residence	Urban	4288	52.68
	Rural	3851	47.32
Occupation	Free businessman	6234	76.60
	Student	642	7.89
	Farmer	326	4.00
	Driver	279	3.43
	Employee	287	3.53
	Policeman	229	2.81
	Military man	142	1.74
Blood group and Rhesus type	O+ve	5510	67.70
	O−ve	435	5.35
	A+ve	1169	14.36
	A−ve	159	1.95
	B+ve	727	8.93
	B−ve	92	1.13
	AB+ve	37	0.46
	AB−ve	10	0.12

**Table 2:** Seroprevalence of the serologic markers of transfusion-transmissible infections (TTIs).

Variable		Seroprevalence, *n* (%)						Total of TTIs: *n* (%)
		Anti-HIV1/2	HBsAg	Anti-HCV	Anti-*T.pallidum*	Serologic Sign of MI	*p* Value	
Year	2016	18 (0.87)	116 (5.60)	22 (1.06)	223 (10.78)	33 (1.60)	˂0.000	412 (19.91)
	2017	76 (2.42)	191 (6.10)	30 (0.96)	358 (11.43)	32 (1.02)	˂0.000	687 (21.93)
	2018	50 (1.70)	187 (6.36)	41 (1.40)	385 (13.10)	71 (2.42)	˂0.000	734 (24.98)
*p* value		˂0.000	0.540	0.256	0.027	˂0.000		˂0.000
Overall		144 (1.77)	494 (6.07)	93 (1.14)	966 (11.87)	136 (1.67)	˂0.000	1833 (22.52)

Statistical differences were evaluated using the Pearson chi-squared test. *n*: number, %: percentage, MI: multiple infections, HIV: human immunodeficiency virus, HBsAg: hepatitis B virus surface Ag, HCV: hepatitis C virus, TTIs: transfusion-transmissible infections.

**Table 3:** Seropositivity rate of the serologic markers of transfusion-transmissible infections (TTIs) in relation to socio-demographic characteristics of blood donors.

Variable		TTIs: *n* (%)	Transfusion-Transmissible Infection: *n* (%)					
			Anti-HIV1/2	HBsAg	Anti-HCV	Anti-*T.pallidum*	Serologic Sign of MI	*p* Value
Age group (Years)	18–27	890 (20.05)	56 (1.26)	209 (4.70)	30 (0.67)	558 (12.57)	37 (0.83)	˂0.000
	28–37	757 (25.38)	64 (2.14)	243 (8.14)	52 (1.74)	331 (11.09)	67 (2.24)	˂0.000
	38–47	186 (25.86)	24 (3.33)	42 (5.84)	11 (1.52)	77 (10.70)	32 (4.45)	˂0.000
*p* value		˂0.000	˂0.000	˂0.000	˂0.000	0.095	˂0.000	
Residence	Urban	654 (15.25)	59 (1.37)	251 (5.85)	38 (0.88)	258 (6.01)	48 (1.11)	˂0.000
	Rural	1179 (30.61)	85 (2.20)	243 (6.31)	55 (1.42)	708 (18.38)	88 (2.28)	˂0.000
*p* value		˂0.000	0.005	0.389	0.022	˂0.000	˂0.000	
Occupation	Free businessman	1462 (23.45)	110 (1.76)	407 (6.52)	78 (1.25)	783 (12.56)	84 (1.34)	˂0.000
	Student	98 (15.26)	8 (1.24)	17 (2.64)	6 (0.93)	56 (8.72)	11 (1.71)	˂0.000
	Farmer	116 (35.58)	10 (3.06)	25 (7.66)	5 (1.53)	62 (19.01)	14 (4.29)	˂0.000
	Driver	56 (20.07)	6 (2.15)	15 (5.37)	2 (0.71)	22 (7.88)	11 (3.94)	˂0.000
	Employee	43 (14.98)	3 (1.04)	12 (4.18)	0 (0)	21 (7.31)	7 (2.43)	˂0.000
	Police man	39 (17.03)	4 (1.74)	12 (5.24)	1 (0.43)	14 (6.11)	8 (3.49)	0.004
	Military man	19 (13.38)	3 (2.11)	6 (4.22)	1 (0.70)	8 (5.63)	1 (0.70)	0.031^f^
*p* value		˂0.000	0.483^f^	0.003	0.408^f^	˂0.000	˂0.000^f^	
Blood group and Rhesus type	O+ve	1295 (23.50)	98 (1.77)	335 (6.07)	71 (1.28)	700 (12.70)	91 (1.65)	˂0.000
	O−ve	94 (21.60)	7 (1.60)	32 (7.35)	8 (1.83)	36 (8.27)	11 (2.52)	˂0.000
	A+ve	241 (20.61)	17 (1.45)	71 (6.07)	8 (0.68)	124 (10.60)	21 (1.79)	˂0.000
	A−ve	46 (28.93)	7 (4.40)	13 (8.17)	2 (1.25)	20 (12.57)	4 (2.51)	˂0.000
	B+ve	124 (17.05)	14 (1.92)	32 (4.40)	4 (0.55)	66 (9.07)	8 (1.10)	˂0.000
	B−ve	21 (22.82)	0 (0)	9 (9.78)	0 (0)	12 (13.04)	0 (0)	˂0.000^f^
	AB+ve	12 (32.43)	1 (2.70)	2 (5.40)	0 (0)	8 (21.62)	1 (2.70)	0.002^f^
	AB−ve	0 (0)	0 (0)	0 (0)	0 (0)	0 (0)	0 (0)	-
*p* value		˂0.000^f^	0.267^f^	0.241^f^	0.260^f^	0.003^f^	0.407^f^	

Statistical differences were evaluated using the Pearson chi-squared and Fisher’s exact tests^f^. *n*: number, %: percentage, MI: multiple infections, HIV: human immunodeficiency virus, HCV: hepatitis C virus, HBsAg: hepatitis B virus surface Ag, TTIs: transfusion-transmissible infections. The degree of variation at statistical level cannot be evaluated for the rate of TTIs among donors carry blood group AB−ve.

**Table 4:** Co-occurrence of the serologic markers of multiple infections.

Variable		Co-Occurrence: *n* (%)	
		Total Donors (*n* = 8139)	Total Donors with Seropositivity for Multiple Markers (*n* = 136)
Type of MI	Anti-*T.pallidum*+Ant-HIV1/2	11 (0.13)	11 (8.09)
	Anti-*T.pallidum*+HBsAg	100 (1.22)	100 (73.53)
	Anti-*T.pallidum*+Anti-HCV	9 (0.11)	9 (6.62)
	Anti-HIV1/2+HBsAg	3 (0.03)	3 (2.20)
	Anti-HIV1/2+Anti-HCV	2 (0.02)	2 (1.47)
	HBsAg+Anti-HCV	5 (0.06)	5 (3.68)
	Anti-*T.pallidum*+Anti-HIV1/2+HBsAg	1 (0.01)	1 (0.73)
	Anti-*T.pallidum*+HBsAg+Anti-HCV	5 (0.06)	5 (3.68)
*p* value		˂0.000	˂0.000

Statistical differences were evaluated by using the Pearson chi-squared test. *n*: number, %: percentage, MI: multiple infections, HIV: human immunodeficiency virus, HCV: hepatitis C virus, HBsAg: hepatitis B virus surface Ag, TTIs: transfusion-transmissible infections.

## Data Availability

Not applicable.

## Supplementary Materials

The following are available online at https://www.mdpi.com/article/10.3390/medicines8110064/s1, Figure S1: Rate of the serologic marker of single infection.
