# Supplementary material for: Seroprevalence of the Serological Markers of Transfusion-Transmissible Infections among Volunteer Blood Donors of Kosti Obstetrics and Gynecology Hospital
Source: Medicines (Basel). 2021 Oct 29;8(11):64. doi: 10.3390/medicines8110064 (PMC8619909; doi:10.3390/medicines8110064)
Supplement: Supplementary file 1 [file medicines-08-00064-s001.zip › medicines-1340443-supplementary.pdf]

medicines

Supplementary Materials: Seroprevalence of the Serological Markers of Transfusion-Transmissible Infections among Volunteer Blood Donors of Kosti Obstetrics and Gynecology Hospital

Babiker Saad Almugadam, Omer Mohammed Ali Ibrahim and Yousif Mousa Alobaid Ahmed

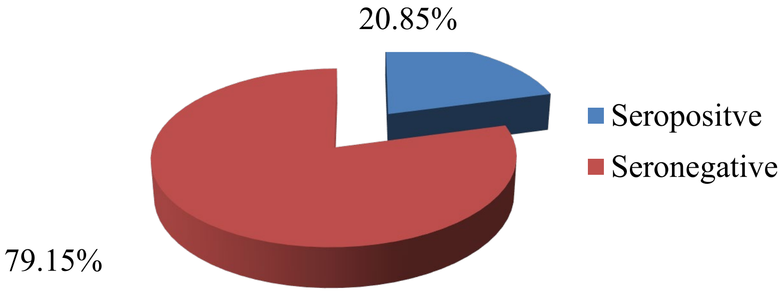

Figure S1. Rate of the serologic marker of single infection.
